# Diversity Assessment of the Montenegrin Maize Landrace Gene Pool Maintained in Two Gene Banks

**DOI:** 10.3390/plants10081503

**Published:** 2021-07-22

**Authors:** Vojka Babić, Violeta Andjelkovic, Zoran Jovovic, Milosav Babic, Vladimir Vasic, Natalija Kravic

**Affiliations:** 1 Maize Research Institute Zemun Polje, Slobodana Bajica 1, 11185 Belgrade, Serbia; violeta@mrizp.rs (V.A.); nkravic@mrizp.rs (N.K.); 2 Faculty of Biotechnology, University of Montenegro, Mihaila Lalića 1, 81000 Podgorica, Montenegro; zoran.jovovic.btf@gmail.com; 3 Institute of Field and Vegetable Crops, Maksima Gorkog 30, 21000 Novi Sad, Serbia; milosav.babic@nsseme.com; 4 Department of Statistics and Mathematics, Faculty of Economics, University of Belgrade, Kamenička 6, 11000 Belgrade, Serbia; vladimir.vasic@ekof.bg.ac.rs

**Keywords:** classification, duplicate accessions, germplasm conservation, morphology, *Zea mays* L.

## Abstract

Due to the loss of agro-biodiversity, there is a strong effort to find apparent and efficient mechanisms for the conservation and sustainable use of genetic diversity. A joint monitoring of the diversity and collections structure of the Montenegrin maize landraces conserved in the Serbian (MRIZPGB) and Montenegrin (MGB) gene banks has been conducted in order to improve the composition of the collections and to identify and eliminate possible redundancy. Based on a separate analysis of white- and yellow-orange maize landraces, it can be concluded that the diversity and evolution of distinct maize landraces grown and collected in Montenegro have been simultaneously shaped by both environmental (i.e., natural selection) and socially driven factors (farmers’ selection, migration and colonization processes of the human population). Although it has been determined that the authenticity and variability of the Montenegrin maize landraces gene pool have largely been preserved in the MRIZPGB collection, a significant amount of redundancy was observed. The obtained results will contribute to the cost-efficient conservation of the maize gene pool in the Montenegrin and Serbian gene banks. The recognized and well-preserved original variability of the MRIZPGB and MGB Montenegrin gene pool represents a valuable source for pre-breeding activities on broadening the white and flint maize breeding programmes under temperate conditions.

## 1. Introduction

Numerous studies underline the importance of accessions conserved in gene banks for their potential use as donors of favourable traits, especially those for overcoming different abiotic and biotic stresses [1,2,3,4,5,6]. A large number of conserved accessions, having been loaded with unfavourable traits, make numerous collections almost useless for breeding and obtaining relevant results in a reasonable period of time, as well as economically unsustainable in terms of their characterization and evaluation [7]. The foundation and maintenance of gene banks are very expensive activities; thus, the application of various strategies for more efficient management is required.

Over 1700 gene banks worldwide conserve over 7 million accessions, out of which only a small portion is fully characterized, with a very limited number being used for crop improvement [8]. A large number of conserved accessions do not even have correct passport data, which also limits their value for potential use.

Numerous problems that make the management and the use of genetic resources more difficult arise from the collection process itself. Many gene banks’ collections started as working collections for taxonomic studies and breeding activities. Afterwards, the collections expanded through collection activities and exchanges with other gene banks. In many cases, this resulted in collections of a considerable size, with an unbalanced composition and an unknown value [9]. Frequently, the collecting process had not been conducted methodically, neglecting data on geographical distribution as well as climate and cultural differences. The accession sample size does not often comply with standardized methodology; hence, rare alleles (alleles of very low frequencies, <0.01), which were present in the population, are not encompassed. Moreover, communication and cooperation among gene banks are often poor [8]. Therefore, collections in gene banks are regularly overcrowded and overloaded with duplicates that occur due to the stated reasons and many others.

The high operational costs of collection maintenance [10] impose the reduction of redundancy as much as possible. Gene banks should not become dead collections that do not fulfil any of their basic missions [11]. The starting criteria for the comparison of accessions are passport data [12], followed by on the evaluation of traits related to kernel and plant morphology [13]. Moreover, molecular markers can contribute effective means towards this goal [14,15,16]. In the study of a large collection, molecular analysis is a highly cost-demanding technique. A possibility to reduce necessary investments is to restrict the molecular screening to the validation of potential duplicate groups determined on the basis of available data sources such as passport data and preliminary morphological characterization/evaluation [17].

The Montenegrin plant gene bank (MGB) in Montenegro, and the Maize Research Institute Zemun Polje Gene Bank (MRIZPGB) in Serbia, have initiated a cooperation with the main goal of studying the diversity and the structure of maize landrace collections, gathered in the territory of Montenegro and conserved in these two institutions.

The aim of the present study was the following: (i) to conduct a detailed analysis of passport data for maize landraces collected in Montenegro and conserved by the MRIZPGB and the MGB; (ii) to classify the MRIZPGB accessions into homogenous groups based on the morphological analysis (with the application of hierarchical grouping models), and later to assign MGB accessions into defined groups (with the application of non-hierarchical grouping models); (iii) to identify possible redundancies based on passport data and the morphological analysis.

## 2. Results

### 2.1. The Analysis of Passport Data

According to the analysis of passport data from the MRIZP gene bank, 64, 225, and 31 accessions were collected in Montenegro during the 1960s, 1970s, and the 1980s, respectively. In relation to the collecting/sponsoring institutions, the Institute of Agriculture Titograd (IAT), the MRIZP, and the Yugoslav Association of Researchers (YAR) together with the United States Department of Agriculture (USDA) collected 136, 124, and 60 accessions, respectively. A pronounced imbalance in terms of the number of collected samples per municipality was observed (Table 1).

A great number of accessions collected in relatively small areas could result in the existence of duplicates, particularly in the municipalities where two different institutions collected a great number of accessions in the same period (e.g., Cetinje, Nikšić and Podgorica). Furthermore, there is an issue of similarities/dissimilarities of accessions collected in the area of the same municipality, in different periods, and by various institutions. For instance, out of 42 accessions collected in the area of Ivangrad, 12 were collected during the 1960s by the YAR and the USDA, 13 during the 1970s, including four accessions collected by the IAT and nine by the MRIZP, whereas the IAT collected another 17 accessions in the same municipality during the 1980s (Table 1).

Sixty-eight maize landraces were collected during seven collecting missions at the MGB through the regional project “South East European Development Network on Plant Genetic Resources (SEEDNet)”, (2004–2010). Two accessions were collected in 2016 (Table 1). According to the MGB accessions passport data, a great portion of accessions (27 out of 70) were collected in only one municipality (Bijelo Polje).

### 2.2. White Maize Landraces

#### 2.2.1. The Classification of MRIZPGB White Maize Landraces Collected in Montenegro

The MRIZPGB collection of white maize landraces was classified into five clusters (CLs I–V) by hierarchical cluster analysis based on 26 standardized phenotypic traits. CL I encompassed early maturing flint landraces with a short plant and small ears. CLs II and V consisted of dent maize landraces with more robust plants, whereas CLs III and IV represent transitional forms (Table 2).

**Figure 1:**
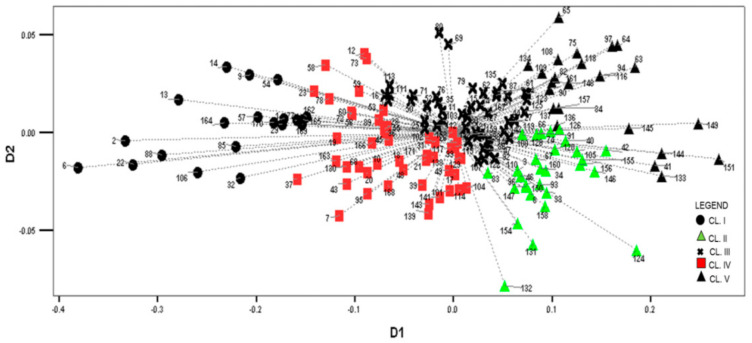
Distribution of MRIZPGB white maize landraces according to the correspondence analysis.

To emphasize the complexity of interrelations among the studied maize landraces, a correspondence analysis was applied according to morphological similarities. The first and the second dimensions of the correspondence analysis encompass 96%, and 4% of inertia (“variability”), respectively. Figure 1 shows the arrangement of landraces in the discriminant space, with cluster classifications labelled by an appropriate mark.

The CL I landraces, with negative values in the first dimension (D1), exhibit the longest vectors and the greatest scattering, especially landraces 6, 2, 22, 13, 14, and 9, collected by the YAR and USDA in 1963. Landraces 57, 170, 29, 77, 169, 162, and 165, collected by the IAT and the MRIZP in territories of various municipalities during the 1970s and the 1980s, are closely positioned to landrace 1, collected by the YAR and the USDA in 1963 (Figure 1). Accordingly, it could be assumed that the earliest collected accessions have the greatest variability, whereas the accessions collected later on most likely originate in these landraces, with an insignificant addition of another germplasm.

The CL II landraces demonstrate positive values for the D1 axis and negative for the D2. The YAR and the USDA collected only two accessions (landraces 3 and 8) in Podgorica and Danilovgrad in 1963 (Figure 1). However, ten years later, the IAT collected one accession (landrace 46) that was similar to landrace 3 and six accessions that were similar to landrace 8, in the territory of the same (landraces 158 and 147) as well as different municipalities (landraces 96, 150, 93, 33), thus pointing to the non-implementation of standard protocols in the collecting procedure. The remaining CL II accessions were collected by MRIZP and IAT in the 1970s, where a number of accessions expressed great similarity. Hence, morphologically similar accessions seem to have been collected by different institutions during the same period (landraces 128, 66, and 126, similar to 74), but also similar accessions have been collected at similar locations by the same or different institutions and in different years (similar to landraces 40, 156, and 105). However, accessions 132, 131, 124, and 154 expressed the highest and most unique diversity.

The differentiation between accessions within CL V, which show positive D1 values, is more pronounced; however, a close positioning of certain accessions can be noticed. These accessions were collected by different institutions in territories of the same or various municipalities in the mid-1970s as follows: accessions 84 (MRIZP) and 157 (IAT), 97 (MRIZP) and 64 (IAT), as well as 82 and 90 (MRIZP), and 148 and 161 (IAT).

The greatest number of accessions belongs to CL III (49) and IV (49), with only seven, i.e., ten accessions, respectively, collected by the YAR and the USDA in 1963. The majority of accessions were collected by the MRIZP and IAT in the 1970s. The accessions of these clusters are placed between CL I (negative values for the first dimension—D1), and CLs II and V (positive values for D1) (Figure 1). Furthermore, a small differentiation between these two clusters is also noticeable. A certain number of accessions within CL III are more similar to the CLs II or V accessions, whereas other accessions are more similar to CL IV accessions. Similarly, some of CL IV accessions are more similar to CL I accessions. Based on the distribution (Figure 1) and average values of morphological traits over clusters (Table 2), it can be stated that these landraces were developed from the crossing of flint (from CL I) and dent germplasm (from CLs II and V), thus representing transitional forms. In addition, CL III accessions, i.e., CL IV accessions, are dent-like, i.e., flint-like. A large overlapping of landraces in these clusters indicates their morphological similarity.

Maize is a cross-pollinated species, and it is very difficult to determine reliably whether two accessions of open-pollinated varieties are duplicates or not. Regardless of the type of information, which is important for decision making (morphological vs. molecular markers), it is very difficult to establish the threshold value that defines the duplicate. Based on the obtained results, it was concluded that collecting activities performed by the YAR and USDA at the beginning of the 1960s were accomplished correctly. Since hybrids were not grown at that time, the 26 accessions of white maize landraces were thought to undoubtedly represent original Montenegrin maize landraces. Based on the distance matrix (Squared Euclidean Distances < 200) of these 26 accessions, a great morphological similarity was established between landraces 6 and 22, and 9 and 14 (CL I), as well as between landraces 15 and 24 (CL III). However, according to the passport data (which included the location and time of collecting, the common name, and the institution that performed the collecting) and the referent gene bank seed collection, these accessions cannot be considered duplicates.

**Figure 2:**
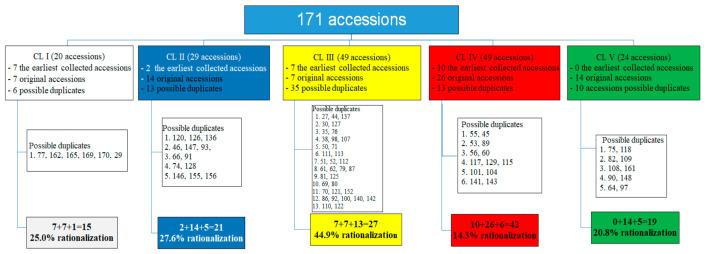
Possible duplicate accessions in the MRIZPGB white Montenegrin maize gene pool.

Regarding the accessions collected subsequently, according to results obtained, the existence of numerous redundant/duplicate accessions can be expected. Based on the performed statistical analyses, distance matrices (Squared Euclidean Distances < 200), passport data, and the referent seed collection of white MRIZPGB maize landraces, a list of possible duplicate accessions for each cluster was made (Figure 2 and Table S1). Accordingly, 25.0, 27.6, 44.9, 14.3, and 20.8% accession redundancies over clusters are observed, respectively, resulting in a 27.5% redundancy in the MRIZPGB Montenegrin white maize pool.

#### 2.2.2. The Allocation of MGB White Maize Landraces into Defined MRIZPGB Clusters

The discriminant analysis showed that 88.3% of originally classified (MRIZPGB) accessions had been correctly classified, i.e., 20 accessions were incorrectly classified by cluster analysis. Obtained probabilities point out that the highest number of incorrectly classified accessions (a total of ten) were placed between CL III and CL IV, indicating a weak differentiation of these two clusters (probability data are not presented).

Since the first discriminant function encompasses 85.5% of variance (Table 3), and the 1000-kernel weight and the kernel width are in the highest correlation with this function (Table 4), the stated traits thus contribute to landrace discrimination to the greatest extent. The majority of the observed traits that correlate with the second discriminant function (Table 4), and which encompass 10.3% of data variability, also significantly affect landrace discrimination. The rest of the traits significantly correlated with the third and the fourth discriminant function (encompassing only 2.3 and 1.9% of variance, respectively) contribute to discrimination to a much lower extent.

Twenty-four MGB white maize accessions were assigned to five existing clusters. The majority of MGB white landraces (a total of 20) were assigned to CL I, which encompassed white, early maturing flints, whereas four landraces were distributed to CLs II, III, IV, and V (68 M, 21 M, 64 M, and 77 M, respectively).

Based on the distance matrix (the squared Euclidean distance < 200), passport data, and ear photos of the MGB white landrace gene pool, it was determined that landraces 13 M and 30 M, and 18 M and 37 M—all belonging to CL I—represent possible duplicates.

**Figure 3:**
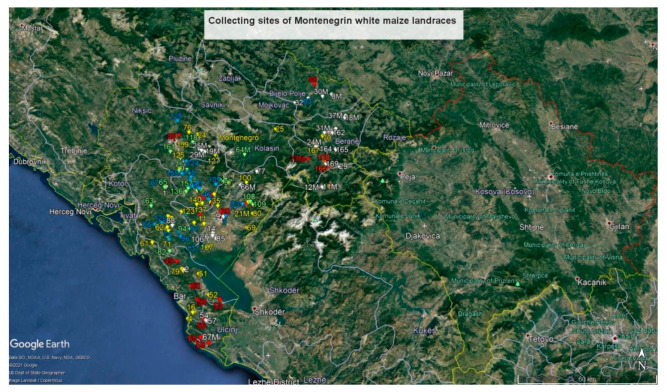
Map of collecting sites for white maize landraces (Maize Research Institute Zemun Polje Gene Bank accessions—designated by number; Montenegrin plant gene bank accessions—designated by number and the letter M; Cluster I—white, Cluster II—blue, Cluster III—yellow, Cluster IV—red, Cluster V—green).

The location of collecting sites for both gene bank accessions is provided in Figure 3 and File S1. Cluster membership is labelled by a different marker colour. On the map, a certain regularity of cluster distribution can be found. The landraces of CL I (white marker) were collected mostly in the southern and eastern part of Montenegro, surrounded by the landraces of CL IV (red marker) and CL III (yellow marker). The landraces of CLs II and V (blue and green markers, respectively) were collected in the central part of Montenegro, at lower altitudes.

### 2.3. Yellow-Orange Maize Landraces

#### 2.3.1. The Classification of Yellow-Orange MRIZPGB Maize Landraces Collected in Montenegro

The grouping of yellow-orange maize landraces was also performed with the creation of five clusters (40, 27, 40, 7, and 13 accessions per cluster, respectively). Based on cluster mean values, flint landraces prevail in each cluster (Table 5).

**Figure 4:**
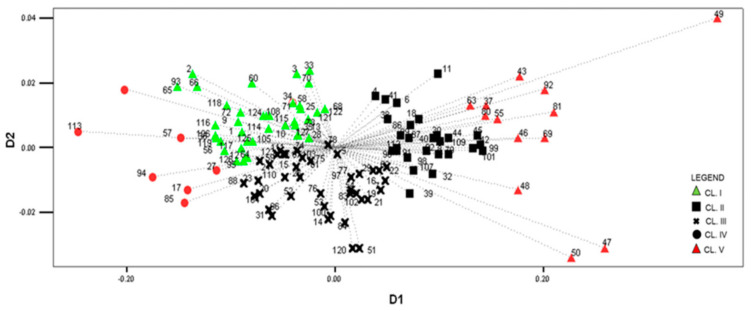
Distribution of MRIZPGB yellow-orange landraces according to the correspondence analysis.

The correspondence analysis showed that the first (D1) and the second (D2) axes encompass 98.0% and 1.7% of inertia (“variability”), respectively. The highest positive values of the D1 dimension in the correspondence analyses (i.e., the longest vectors), were recorded for the CL V accessions (Figure 4). These landraces are the early flints (FAO 200), with the most robust plant, the lowest number of kernel rows (11.01), and the largest kernels (Table 6). According to passport data, all these landraces were collected in the coastal parts of Montenegro by the IAT (six accessions) and the MRIZP (seven accessions) in mid-1970s.

Less pronounced positive values of the D1 dimension (i.e., shorter vectors) were detected in the CL II accessions. In comparison with CL V landraces, these landraces are also flints, but of medium-early maturity (FAO 300), with somewhat smaller kernels and a not-so-robust plant. Only five accessions (4, 6, 8, 11, and 18) were collected by the YAR and the USDA in 1963. The rest (23 accessions) were collected by the MRIZP and the IAT in the mid-1970s, mostly in the Nikšić municipality, where numerous landraces express a great morphological similarity. Hence, the existence of possible duplicates among these landraces is a consequence of the poor coordination of collecting activities among different institutions, as well as the poor implementation of a standard collecting protocol.

The CL IV accessions had extremely negative values for the D1 dimension. CL IV encompasses the smallest number of landraces, with the hardest and the smallest orange kernels, with the shortest plants and 14 kernel rows per ear (Table 6). Only one landrace was collected in 1963 (Plav municipality), while the other six were collected during the 1970s (Plav, Nikšić, Kolašin, and Mojkovac municipalities). These are extra-early maturing landraces that were grown at higher altitudes (800–1100 m above sea level). There is no overlapping of landraces, thus indicating their morphological distinctness (Figure 4).

The CL I accessions have negative values for D1, while the CL III accessions have negative values for the D2 dimension (Figure 4). The highest number of landraces was encompassed by these two clusters (40 each), with a large number of morphologically similar landraces per cluster. A close examination of the passport data for the CL I accessions shows that similar landraces (e.g., 3, 33, and 70; or 2, 66, and 93) were mostly collected during the same collecting periods, by the same/different institution(s), at different locations. An opposite trend, i.e., the collection of morphologically similar landraces at the same location regardless of the collecting institution(s) and period was also found. A similar trend was found for passport data and the distribution of CL III landraces. Some of the CL III landraces are morphologically more similar to CL I and to CL II accessions (Figure 4). Even the earliest collecting missions conducted by the YAR and the USDA in 1963, collected at different locations, resulted in morphologically similar accessions (7, 16, 19, 21, and 22).

Twenty-two yellow-orange landraces (7, 5, 9, 1, and 0 per cluster, respectively) collected by the YAR and the USDA in the 1960s can be considered as original Montenegrin maize landraces. These landraces were collected first and there is a lower probability of duplicate occurrence. Although closely positioned in Figure 4, landraces 7, 1, and 19 cannot be considered as duplicates according to distance matrix, passport data, and the referent seed samples. Based on the distance matrix, landraces 2 and 16 were very similar to landraces 93 and 62, which were collected much later, although the assumption of being duplicates was rejected based on the passport data and the referent seed samples.

**Figure 5:**
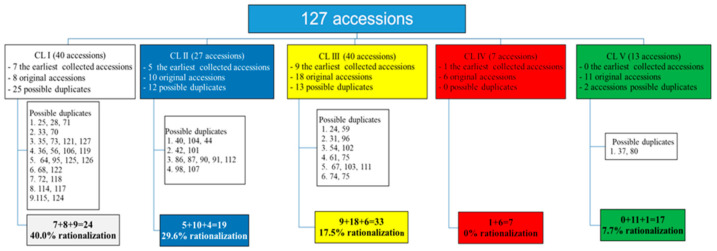
Possible duplicate accessions in the MRIZPGB yellow-orange Montenegrin maize gene pool.

In comparison to early collecting missions, subsequent ones produced a certain number of duplicate accessions that were collected by the MRIZP and IAT in addition to the original accessions. Based on the distance matrix, passport data, and the referent seed collection, the accessions that could be considered duplicates within each cluster were identified (Figure 5 and Table S2). The average portion of redundant accessions in the MRIZPGB Montenegrin yellow-orange maize pool was estimated at 25.7%. Portions of highly likely duplicate accessions were 40.0, 29.6, 17.5, 0.0, and 7.7% for CLs I to V, respectively.

#### 2.3.2. The Allocation of MGB Yellow-Orange Maize Landraces into Defined MRIZPGB Clusters

The discriminant analysis showed that 94.5% of yellow-orange MRIZPGB accessions were correctly classified by the cluster analysis, while the weakest discrimination was between the CL I and CL III clusters. Seven accessions were misclassified. Eigenvalues (Table 6) show that the first two discriminant functions encompass 93.0% of variability (79.2% and 13.8%, respectively). The traits with significant correlations to the first as well as to the second function (Table 7) contributed the most to landrace discrimination.

Out of the total number of MGB accessions, 46 are yellow-orange landraces. The majority of the MGB accessions were designated to CL IV (32) and CL III (11), while only one accession for CLs I and II was assigned. Not a single accession was designated to CL V.

The distance matrix, passport data, and photos of ears determined the absence of duplicate accessions within the CL III gene pool of the MGB yellow-orange maize landraces. Three pairs of possible duplicates were determined within the CL IV accessions (6 M and 14 M; 9 M and 10 M; 26 M and 57 M). Even though almost half of these accessions were collected in one municipality, only one pair of possible duplicates (26 M and 57 M) was identified.

**Figure 6:**
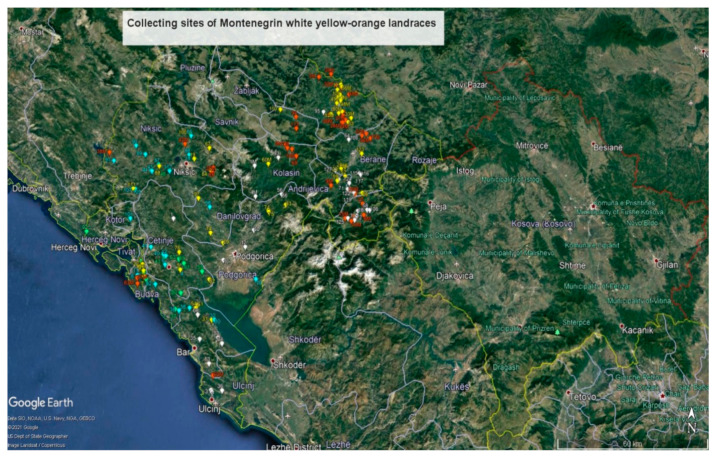
Map of collecting sites for yellow-orange maize landraces (Maize Research Institute Zemun Polje Gene Bank accessions—designated by number; Montenegrin plant gene bank accessions—designated by number and the letter M; Custer I—white, Cluster II—blue, Cluster III—yellow, Cluster IV—red, Cluster V—green).

The locations of collecting sites of the MRIZPGB and the MGB yellow-orange maize accessions are presented on the map of Montenegro (Figure 6 and File S1). The largest number of MGB accessions was collected in the northern and eastern parts of Montenegro. The MRIZPGB accessions were collected in the southern and south-western parts of the country, and also a certain number was collected in central parts of Montenegro. The geographical distribution of the clusters on the map is in accordance with the results of the correspondence analysis. Particularly, landraces belonging to CLs I, III, and IV, collected predominantly in the eastern and north-eastern parts of Montenegro, have negative values for the D1 axis, while CLs II and V landraces, collected in southern and south-western parts of the country, have positive values for the D1 axis. It could be concluded that a more frequent exchange of genetic material in nearby cultivation areas resulted in a high level of morphological similarity among these groups of landraces.

## 3. Discussion

### 3.1. The Analysis of Passport Data

Since its foundation (1945), the MRIZP breeding programme has included the collecting of local maize populations as well as the introduction of maize germplasm from abroad. In time, the gene bank for maize has established its own comprehensive research programme, using appropriate scientific methods and adequate facilities, and thus affirming itself as the national Yugoslav gene bank for maize. Nowadays, the MRIZP gene bank conserves 2217 accessions of maize landraces from the former Yugoslavia, including 320 landraces collected in Montenegro [18,19].

The initial collecting missions by autochthonous populations of agricultural plants in Montenegro were performed at the end of the 1940s, exclusively for scientific purposes. The implementation of the SEEDNet Project allowed for the establishment of the Montenegrin plant gene bank—MGB in 2004. Currently, the MBG conserves 70 maize landraces and very little has been done so far to study the maize gene pool in Montenegro [20]. Therefore, a cooperation between the MRIZPGB and MGB has been established with the aim of comparing the two existing collections from the aspect of their diversity. The identification of possible duplicates is often started by examining the passport data [21,22]. Prior to comparisons of different collections, an in-house analysis of diversity and the identification of redundancies facilitate gene bank management [23].

The hilly/mountainous regions of Montenegro, where agricultural production is performed in small and isolated fields, is favourable for great diversity of maize populations. A close study of the passport data of the MRIZPGB accessions shows that the organised collecting missions began at the right time (i.e., prior to introduction of hybrid varieties into production), but also that the large number of accessions were collected in a relatively small area, which could generate the existence of numerous redundant accessions. These findings are in line with the fact that the various institutions collected a very unbalanced number of accessions in the territories of the same municipality (even in the same period), as a consequence of poor planning and coordination of collecting activities [24]. On the other hand, the MGB accessions passport data indicated that the organised collecting missions were initiated quite late and that the large number of accessions (27) was collected in the territory of only one municipality (Table 1). This is most likely a consequence of the depopulation of Montenegrin rural regions in the 20th and 21st centuries, which has led to the fact that maize is no longer grown in many areas where it used to be grown, which is in agreement with studies on different species [25].

The large number of accessions and the presumed high level of duplication, both within and between collections, burdened the efficient use of existing variability. Given the similar issues, many gene banks have become more concerned with the assessment of diversity within their collections and with the improvement of the efficiency of genetic resource management, than with the acquisition of new material [26,27]. On the other hand, the passport data point to many vernacular names with common terms, so potential duplicates based on passport data need to be validated by additional data in order to avoid wrong decisions. In that context, molecular marker technologies are increasingly being used to assess diversity [14,16] or to validate redundancy. Absolute certainty that two samples are identical can only be inferred from a DNA sequence comparison of their entire genomes [9]. Consequently, the analyses are restricted to a limited number of markers, which should preferably be used in conjunction with passport, morphological, and evaluation data [28]. However, in a study of a large collection, as was the case in this study, molecular analysis proved to be a highly cost-demanding technique. Therefore, the analysis and comparison of existing MRIZPGB and MGB accessions were initially done at the morphological level. Moreover, white and yellow-orange landraces were analysed separately due to their different genetic background, formed through long-term separate cultivation and for different purposes [29,30].

### 3.2. White Maize Landraces

On the map of white landraces collecting sites (Figure 3), sites in the southern and eastern parts of Montenegro prevailed. This is partly in accordance with [31,32], that white maize were mainly cultivated in the south and central parts, while yellow landraces were cultivated in the eastern and north-eastern areas of the country. Such regional distribution of local genotypes persist until today, but with significantly smaller cultivation. It can be seen that CL I landraces are positioned mostly at higher altitudes, along the border with Albania and Kosovo, which is inhabited by ethnic Albanians and Bosniaks [33] who have been living quite isolated from the Serbian and Montenegrin population. Orozco-Ramirez et al. [34] set the hypothesis that ethno-linguistic differences could affect the diversity of landraces, sometimes even more than edaphic and climatic environmental conditions. Therefore, it can be assumed that under conditions of ethno-linguistic barriers, a specific variability of CL I landraces was developed and preserved. The CL IV landraces, which are morphologically most similar to CL I landraces (Figure 1), have mainly been collected in the vicinity of the CL I landraces’ collecting sites at slightly lower altitudes. Comparing highland and lowland maize landraces from four east-central Mexican states, [35] reported the effect of altitude on genetic differentiation. The first maize introduction to the Balkan region occurred with flint types from the Caribbean islands, the Mexican plateau, and the Andes during the 16th century [36,37], and it is possible that the original variability had been preserved in these two clusters. Pavićević [31] stated that Montenegrin and Mediterranean flints and Kosmet flinty-dents are considered to be the eldest types of maize in the Balkans. The collecting sites of CL III landraces are often close to those of CL IV landraces (Figure 3). These landraces, intermediate in their morphological properties (Figure 1, Table 3), are probably a mixture of the germplasm of the first introductions of flints and the later introductions of dents. Pressior and Berthaud [38] argued that cultivation in different villages and selection by farmers contributed to morphological differentiation, but that pollen migration among populations reduced genetic separation.

Most of the CLs V and II landraces (dents) grown at 450 m, i.e., 250 m above sea level, was collected in the central part of Montenegro. CL II landraces are characterised by the tallest plants, while CL V landraces have the largest kernels (Table 2). Interestingly, the first collection performed by the YAR and USDA in the 1960s included only two white dent landraces assigned to CL II; the remaining fifty-one were collected by IAT and MRIZP, mostly in the 1970s. It is not possible to be absolutely sure whether these types of white maize landraces were first grown in Montenegro in the 1960s, or whether similar ecotypes had already been collected in sufficient numbers at other locations of the former Yugoslavia. Therefore, the collection of flints was emphasised as they were less distributed in other parts of the former Yugoslavia. Studies of Babić et al. [30] highlight the great morphological similarity of two in situ landraces grown in western Serbia with the landrace 84 (CL V) from Montenegro. The spreading of dent maize landraces from Serbia across Montenegrin lowlands most likely occurred through the exchange of seeds among families. Orozco-Ramirez et al. [33] stated that studies on seed exchange/trade paths could significantly contribute to understanding the processes that pattern genetic diversity in maize.

Obtained results indicated that a large number of similar accessions within the MRIZPGB white maize gene pool were found in CLs III, IV, and II. Suggested potential redundancies according to passport and morphological data should be validated with molecular markers [14]. When groups of duplicates have been validated and redundancy identified, it has to be decided which accession from each group of duplicates should be maintained in the collection and which accessions should be discarded, or possibly merged. The loss of rare alleles could be avoided through the lumping of accessions, appropriate for the out-crossing of species such as that performed with maize [39].

The poorest discrimination was between the CLs III and IV landraces. The vicinage of collecting sites for accessions within these clusters indicates a possible common origin and a significant exchange of genetic material via pollen. Similar to reports of Salami et al. [40], the discriminant analysis recognised the most discriminating variables, i.e., traits that contributed the most to cluster differentiation (Table 3 and Table 4).

The assignment of white MGB accessions into CL I—early flints, indicated far less variability of these landraces compared to the Montenegrin landraces conserved in the MRIZPGB collection. Although significant efforts have been made within the SEEDNet Project on collecting and conserving maize landraces, this study supports the assertion that the preservation of the maize gene pool in Montenegro was approached with a considerable delay [41]. The rural depopulation in Montenegro, the introduction of hybrid varieties into agricultural production, and the lack of clear plans for landrace conservation have led to the loss of many indigenous maize landraces. Fortunately, this study also confirms that much of the variability has been preserved within the MRIZPGB collection in Serbia. The most original variability has certainly been determined in the MRIZPGB accessions collected in the early 1960s, but the variability of dent type accessions collected later is also considered to be significant.

### 3.3. Yellow-Orange Maize Landraces

In the collection of yellow-orange MRIZPGB maize landraces, accessions with the flint type predominate. According to Pavićević [31], the highest number of landraces collected in Montenegro belongs to the genetically relatively pure Montenegrin flints, while a smaller number has certain characteristics of dents and semi-dents, being a consequence of their cultivation close to modern selections.

A clear differentiation of CLs IV and V accessions in the correspondence analysis plot indicates the authentic morphology of these landraces, which is opposite to numerous overlapping of landraces belonging to the remaining clusters (Figure 4). According to morphological similarities and passport data, it can be concluded that there was a poor coordination of collecting activities among various participants, especially between the MRIZP and the IAT. Gene banks collecting activities were often performed on the principle of “grab bag” and a non-compliance with the standards for collection establishment [42]. In order to avoid this, significant efforts have been made to identify duplicates and/or rare accessions with unique alleles [8].

A great concordance is observed on the map comparing collecting sites (Figure 6) along with the clustering results (Figure 4). It can be assumed that landraces with large flinty kernels and the largest habitus (CL V accessions), collected at low altitudes in the coastal region of Montenegro, have spread to the north and west; through the process of selection and adaptation, landraces with shorter plants and smaller kernels (CL II accessions), cultivated at slightly higher altitudes, were thus created over time.

In relation to CLs V and II, an opposite spatial distribution was found for the CL IV and I accessions (Figure 4 and Figure 6); this is in line with findings of Vega-Alvarez et al. [43], that geographic origin affected population differentiation and dispersion. These landraces have been cultivated along the Albanian border, and similar to CL I of white maize landraces, their distribution corresponds to the territory inhabited by ethnic Albanians and Bosniaks. Therefore, the specificity of these groups of landraces could be related not only to edaphic-climatic specificities, but also to ethno-linguistic barriers [33].

The central position of CL III accessions (Figure 4 and Figure 6) indicated that they represent transitional forms of the opposite groups. These landraces, intermediate in numerous phenotypic traits, have probably been developed by mixing the germplasm of opposite clusters.

Within the MRIZPGB collection, a large number of Montenegrin indigenous maize landraces are early maturing flinty landraces, and as such, they are carriers of traits important for overcoming different types of biotic and abiotic stresses. Studies performed by Babić et al. [44,45] and Popović et al. [46] showed that among maize landraces in the Western Balkans, identified as a source of tolerance to drought, almost one-third is of Montenegrin origin. Moreover, due to the specificity of the terrain, maize cultivation on the small, isolated fields, and the extensive method of production, it could be assumed that the Montenegrin landraces had preserved the authenticity of the first populations introduced to the Balkans [32].

The assignment of the MGB accessions into existing groups indicated that the majority of accessions were classified into CLs III and IV. The landraces similar to CL V accessions no longer exist, while landraces similar to CLs I and II landraces are on the edge of the extinction. It is most probable that the numerous ecotypes ceased to be cultivated due to rural depopulation, and due to the transition to the cultivation of hybrid varieties [47]. In addition, a disproportionately large number of accessions collected in one municipality (Table 2) suggests that the cultivation of yellow-orange OPVs (used for feed) was maintained only at higher altitudes in a small number of households and under conditions of extensive agricultural production. Pavićević [32] found that the lack of financial resources, technical facilities, and clear conservation plans in Montenegro resulted in the complete loss of numerous autochthonous maize landraces. Similar conclusions were drawn by Tenaillon and Sharcosset [48], who reported the expansion of high-yielding hybrids initiated after the Second World War, which completely altered the process of maize cultivation.

## 4. Materials and Methods

### 4.1. Field Experiments

The morphological characterisation of 320 MRIZPGB accessions was conducted according to the CIMMYT/IBPGR Descriptors for Maize [49] in 2015 and 2016; 298 were successful. Characterisation of 70 MGB accessions was successfully performed in 2017 and 2018. Traits, developmental stages of plants, as well as the minimum number of plants or plant parts necessary for evaluation and methodology are defined in the Descriptors. Landraces were sown in two rows, with 20 plants per row, in two replications, using standard cropping practices at the location of Zemun Polje, Serbia (44°52′ N, 20°19′ E, 81 m asl). According to European Environmental Stratification [50], the experimental sites were assigned to the Pannonian 3 (PAN3) zone within a temperate continental climate. The soil type was a slightly calcareous chernozem. Within each replication, a total of twenty plants per landrace were analysed.

The following 26 phenotypic traits were evaluated: plant height, ear height, number of leaves above the ear, total number of leaves, leaf length, leaf width, tassel length, tassel peduncle length, length of the branching part of tassel, number of primary tassel branches, number of secondary tassel branches, kernel row number, number of kernels per row, ear length, ear width at the top of ear, ear width in the middle of ear, ear width at the base of ear, cob width, rachis width, kernel length, kernel width, kernel thickness, kernel hardness, kernel dentiness, and 1000-kernel weight.

### 4.2. Statistical Analyses

Maize landraces differing in kernel colour express distinct properties, genetic background, and utilisation value [51]. Therefore, the analysis of white (171 accessions) and yellow-orange (127 accessions) MRIZPGB Montenegrin landraces was performed separately. Standardized morphological traits were used for the hierarchical cluster analysis [52]. The squared Euclidean distance was calculated as a measure of the distance, whereas complete-linkage clustering was used as a method of grouping. To emphasise the complexity of interrelations between studied maize landraces, a correspondence analysis was performed according to morphological similarities and results were graphically presented [53,54]. Its advantage is that it does not assume the assignment of units into certain groups and presents continued variability more precisely, which is especially important in cases when significant genetic exchange between geographically close populations is present [55].

After the definition of homogenous groups of the MRIZPGB accessions by cluster analysis, 70 MGB accessions were assigned to the appropriate cluster using a discriminant analysis [56], which allowed for both the verification of quality of the MRIZPGB accessions’ clustering and the proper assignment of MGB accessions into defined clusters. The statistical package IBM SPSS Statistics 25 was used for data analysis.

## 5. Conclusions

The authenticity and variability of the Montenegrin maize landrace gene pool have largely been preserved due to collecting activities initiated prior to the introduction of hybrid varieties into commercial production. A higher genetic variability of the Montenegrin maize landrace gene pool was observed in the MRIZPGB collection. Diversity and the evolution of landraces have been simultaneously shaped by both environmental (i.e., natural selection) and socially driven factors (selection by farmers, migration, and colonisation processes of the human population). Identified possible duplicates within the MRIZPGB and MGB collections will contribute to the rationalisation of conservation activities for maize genetic resources in Serbia and Montenegro; these will also promote the performance of more efficiently planned studies on the genetic variability and structure of the Montenegrin maize landrace gene pool by molecular markers. The direct incorporation of genetic resources into breeding programmes is inefficient due to the lack of the possibility to collect all the desirable characteristics from a small number of generations while maintaining all the positive characteristics of the elite material. The ambition of the cooperation between MRIZPGB and MGB is to start and speed-up the mobilization of currently scattered, unrecognized, and underused maize genetic resources maintained in two collections, as well as facilitate their use in pre-breeding activities in order to make them suitable for the broadening of the base of white and flint commercial maize breeding programmes under temperate conditions.

## Figures and Tables

**Table 1:** Passport data of the MRIZPGB and MGB maize landraces collected in Montenegro.

Municipality of Collecting Site	MRIZPGB							MGB			
	Collecting/Sponsoring Institutions										
	YAR and USDA	IAT		MRIZP				MGB			
	Period of Acquisition										
	‘60s	‘70s	‘80s	‘60s	‘70s	‘80s	Σ	2009	2010	2016	Σ
Bar	2	12		1	6		21	1			1
Cetinje	6	11			26		43				
Danilovgrad	8	34		1	5		48				
Ivangrad	12	4	17		9		42				
Kolašin	1				4		5	6			6
Kotor	3	5		2			10	1	1		2
Nikšić	4	10	4		19		37	7			7
Podgorica	13	15			32		60	2	1		3
Ulcinj	4	8					12	2			2
Plav		4	8		7		19	3	1		4
Bijelo Polje		1			8		9	23	4		27
Budva		2			1		3	1			1
Herceg Novi					1		1				
Mojkovac			1		1	1	3				
Unknown	7						7				
Berane								6	1	1	8
Gusinje								4			4
Andrijevica								2	1	1	4
Pljevlja								1			1
Σ	60	106	30	4	119		320	59	9	2	70

MRIZPGB—Maize Research Institute Zemun Polje Gene Bank; MGB—Montenegrin plant gene bank; YAR—Yugoslav Association of Researchers; USDA—United States Department of Agriculture; IAT—Institute of Agriculture Titograd; MRIZP—Maize Research Institute Zemun Polje.

**Table 2:** Cluster mean values for the measured traits of MRIZPGB white maize landraces.

Trait	Cluster				
	CL I	CL II	CL III	CL IV	CL V
Plant height	149.51	210.77	174.89	177.87	184.76
Ear height	45.53	78.48	60.66	62.23	63.58
NLAE	4.60	5.46	5.20	5.18	5.15
NL	13.33	16.10	14.58	14.78	14.67
Leaf length	56.17	76.20	64.25	66.64	69.56
Leaf width	7.56	8.62	8.08	8.03	8.15
Tassel length	46.67	59.71	52.05	52.47	55.41
TPL	19.63	22.68	20.97	21.00	21.51
LBT	27.04	37.02	31.07	31.46	33.90
NPTB	16.95	17.34	16.87	16.93	17.20
NSTB	4.41	4.42	4.27	4.45	4.48
NKR	11.36	10.98	10.74	10.78	10.01
KPR1	23.09	31.75	25.23	26.71	27.18
KPR2	23.90	32.38	25.96	27.19	27.94
Ear length	11.32	14.66	12.72	12.75	13.79
Ear diameter—tip	3.00	3.61	3.32	3.19	3.49
Ear diameter—middle	3.52	4.14	3.90	3.70	4.06
Ear diameter—basis	3.89	4.51	4.32	4.08	4.47
Cob diameter	2.06	2.23	2.22	2.04	2.26
Rachis diameter	1.72	1.90	1.89	1.72	1.91
Kernel length	0.90	1.10	0.99	0.98	1.05
Kernel width	0.87	1.02	0.99	0.94	1.08
Kernel thickness	0.46	0.45	0.46	0.45	0.49
Kernel hardness	3.38	2.46	3.01	2.98	2.66
Kernel dentiness	2.56	3.40	2.92	2.88	3.25
1000 kernels weight	268.90	380.07	360.98	321.22	414.63

NLAE—number of leaves above the ear; NL—total number of leaves; TPL—tassel peduncle length; LBT—length of the branching part of tassel; NPTB—number of primary tassel branches; NSTB—number of secondary tassel branches; NKR—number of kernel rows; KPR—kernels per row.

**Table 3:** Eigenvalues and % of variance for discriminant functions.

Function ^1^	Eigenvalue	% of Variance	Cumulative %	Canonical Correlation
1	7.642	85.5	85.5	0.940
2	1.002	10.3	95.8	0.693
3	0.202	2.3	98.1	0.410
4	0.174	1.9	100.0	0.385

^1^ First four canonical discriminant functions were used in the analysis.

**Table 4:** Pooled within-groups correlations between discriminant variables and standardized canonical discriminant functions.

Trait ^1^	Function			
	1	2	3	4
1000 kernels weight	0.910 *	−0.025	−0.059	−0.011
Kernel width	0.491 *	−0.018	0.090	−0.277
Plant height	0.267	0.772 *	0.153	0.191
Ear height	0.229	0.716 *	−0.053	0.217
Leaf length	0.227	0.629 *	0.140	−0.134
Total number of leaves	0.156	0.581 *	−0.095	0.196
Kernels per row1	0.140	0.571 *	0.310	0.030
Kernels per row2	0.141	0.539 *	0.338	0.065
Tassel length	0.199	0.466 *	0.193	0.048
Kernel length	0.242	0.434 *	0.272	0.193
Length of the branching part of tassel	0.189	0.433 *	0.205	0.016
Ear length	0.195	0.387 *	0.227	0.004
Leaf width	0.129	0.332 *	−0.003	0.228
Kernel hardness	−0.128	−0.245 *	−0.169	0.045
Kernel dentiness	0.157	0.238 *	0.221	0.013
Kernel thickness	0.073	−0.231 *	0.191	−0.223
Tassel peduncle length	0.078	0.196 *	0.043	0.061
Number of leaves above the ear	0.125	0.321	−0.389 *	0.122
Number of prim. tassel branches	0.013	0.033	0.097 *	−0.007
Ear diameter—middle	0.244	0.170	0.198	0.386 *
Ear diameter—basis	0.236	0.106	0.090	0.381 *
Number of kernel rows	−0.080	0.100	0.113	0.378 *
Cob diameter	0.110	−0.077	0.105	0.364 *
Rachis diameter	0.102	−0.053	0.066	0.335 *
Ear diameter—tip	0.235	0.268	0.250	0.282 *
Number of secondary tassel branches	0.000	0.022	0.065	−0.113 *

^1^ Variables ordered by absolute size of correlation within function; * Largest absolute correlation between each variable and any discriminant function.

**Table 5:** Clusters’ mean values of the measured traits for the MRIZPGB yellow-orange maize landraces.

Trait	Cluster				
	CL I	CL II	CL III	CL IV	CL V
Plant height	138.97	175.41	179.00	137.34	185.17
Ear height	39.02	60.75	64.72	38.62	70.23
NLAE	4.62	5.27	5.07	4.46	5.10
NL	12.86	14.56	14.45	12.30	15.31
Leaf length	52.3	66.3	65.49	56.36	66.72
Leaf width	7.28	8.08	7.93	6.97	8.13
Tassel length	44.02	53.29	52.74	44.27	53.49
TPL	18.67	20.98	21.78	18.07	20.69
LBT	25.35	32.31	30.96	26.20	32.80
NPTB	14.32	18.05	17.27	13.39	17.65
NSTB	3.42	4.88	4.24	2.87	4.34
NKR	12.39	11.9	12.80	13.40	11.01
KPR1	20.12	25.69	26.08	22.08	23.47
KPR2	20.86	26.19	26.94	22.28	24.30
Ear length	9.67	13.64	12.84	9.59	13.06
Ear diameter—tip	2.97	3.44	3.28	2.94	3.39
Ear diameter—middle	3.49	3.99	3.79	3.37	4.05
Ear diameter—basis	3.83	4.43	4.16	3.65	4.60
Cob diameter	2.06	2.33	2.2	2.00	2.43
Rachis diameter	1.71	2.00	1.85	1.68	2.04
Kernel length	0.88	0.96	0.94	0.86	0.99
Kernel width	0.84	0.95	0.86	0.77	1.03
Kernel thickness	0.48	0.49	0.47	0.44	0.54
Kernel hardness	3.65	3.36	3.53	3.67	3.36
Kernel dentiness	2.34	2.58	2.46	2.30	2.62
1000 kernels weight	259.13	352.74	281.41	198.86	409.08

NLAE—number of leaves above the ear; NL—total number of leaves; TPL—tassel peduncle length; LBT—length of the branching part of tassel; NPTB—number of primary tassel branches; NSTB—number of secondary tassel branches; NKR—number of kernel rows; KPR—kernels per row.

**Table 6:** Eigenvalues and % of variance for discriminant functions.

Function ^1^	Eigenvalue	% of Variance	Cumulative %	Canonical Correlation
1	8.518	79.2	79.2	0.946
2	1.490	13.8	93.0	0.774
3	0.611	5.7	98.7	0.616
4	0.140	1.3	100.0	0.351

^1^ The first four canonical discriminant functions were used in the analysis.

**Table 7:** Pooled within-group correlations between discriminant variables and standardised canonical discriminant functions.

Trait ^1^	Function			
	1	2	3	4
1000 kernels weight	0.848 *	0.211	−0.001	0.035
Kernel width	0.441 *	0.136	−0.035	0.026
Plant height	0.376 *	−0.172	0.130	0.009
Ear height	0.334 *	−0.237	0.062	0.091
Leaf length	0.263 *	−0.169	0.168	0.068
Total number of leaves	0.226 *	−0.083	0.056	−0.107
Kernels per row1	0.219 *	−0.126	−0.029	−0.078
Kernels per row2	0.121 *	−0.028	0.015	−0.083
Tassel length	−0.117 *	0.031	0.033	0.101
Kernel length	0.191	−0.569 *	0.130	0.012
Length of the branching part of tassel	0.194	−0.567 *	0.210	0.071
Ear length	0.344	−0.540 *	0.500	−0.006
Leaf width	0.293	−0.533 *	0.236	−0.279
Kernel hardness	0.339	−0.514 *	0.089	0.189
Kernel dentiness	0.288	−0.488 *	0.285	0.035
Kernel thickness	0.295	−0.405 *	0.190	−0.181
Tassel peduncle length	0.271	−0.333 *	−0.020	0.162
Number of leaves above the ear	0.107	−0.291 *	0.225	0.265
Number of prim. tassel branches	0.169	0.264 *	0.196	0.094
Ear diameter—middle	0.167	−0.237 *	0.128	0.213
Ear diameter—basis	−0.124	−0.170 *	0.076	−0.067
Number of kernel rows	0.303	−0.468	0.529 *	−0.109
Cob diameter	0.253	−0.226	0.352 *	0.020
Rachis diameter	0.141	−0.177	−0.067	0.371 *
Ear diameter—tip	0.200	−0.218	0.109	0.248 *
Number of secondary tassel branches	0.171	−0.215	0.052	0.218 *

^1^ Variables ordered by absolute size of correlation within function; * Largest absolute correlation between each variable and any discriminant function.

## Data Availability

Passport data for MRIZPGB and MGB were transferred to the EURISCO Database https://eurisco.ipk-gatersleben.de/ (accessed on 20 May 2021).

## Supplementary Materials

The following are available online at https://www.mdpi.com/article/10.3390/plants10081503/s1, File S1: Collecting sites of Montenegrin maize landraces (animation is active on mouse click), Table S1: Passport and morphological characterization data for potential duplicates within white maize landraces gene pool, Table S2: Passport and morphological characterization data for potential duplicates within yellow-orange maize landraces gene pool.
