# Supplementary figures and images for: Diversity Assessment of the Montenegrin Maize Landrace Gene Pool Maintained in Two Gene Banks
Source: Plants (Basel). 2021 Jul 22;10(8):1503. doi: 10.3390/plants10081503 (PMC8399334; doi:10.3390/plants10081503)

## Slide 1
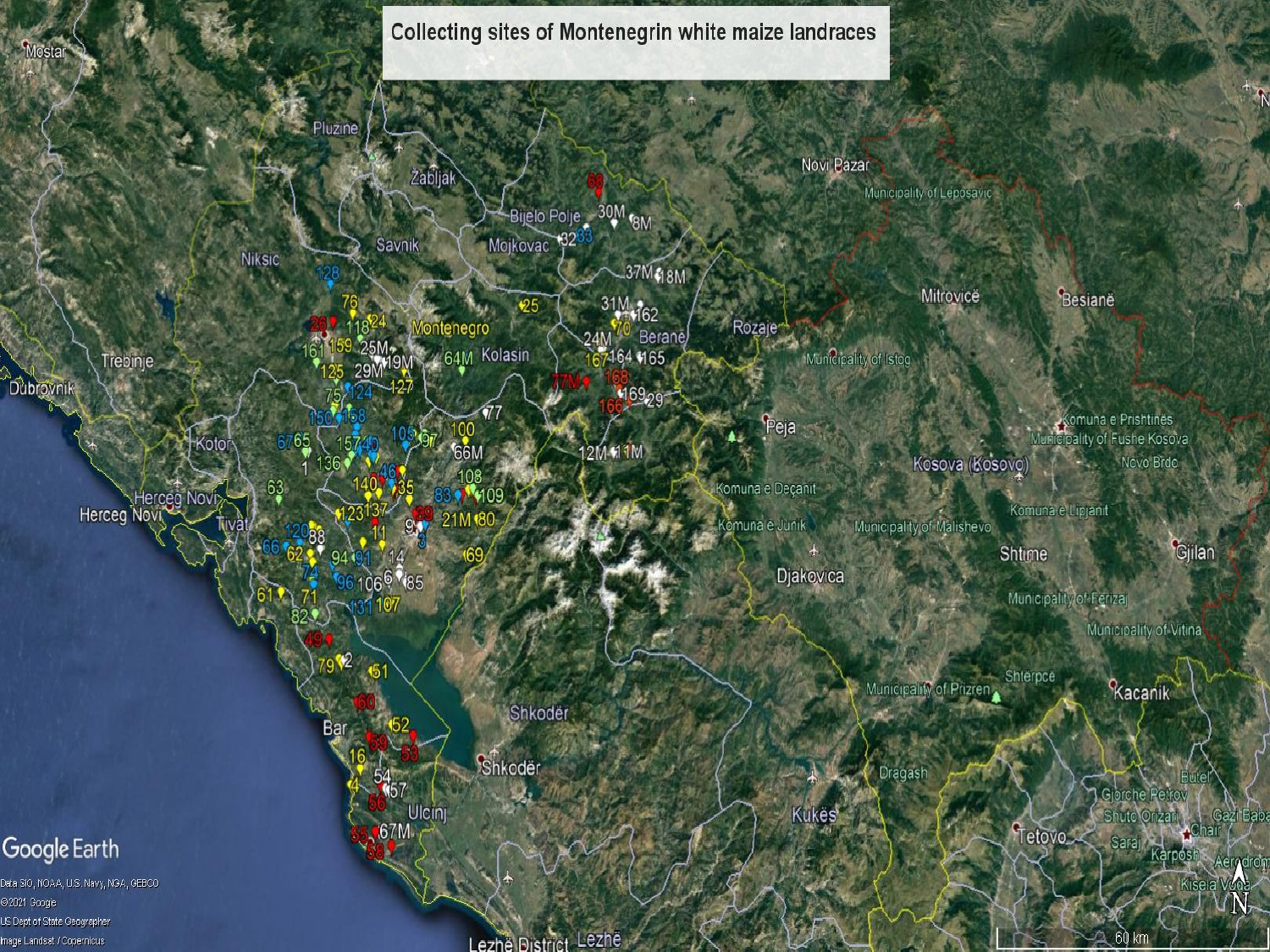

## Slide 2
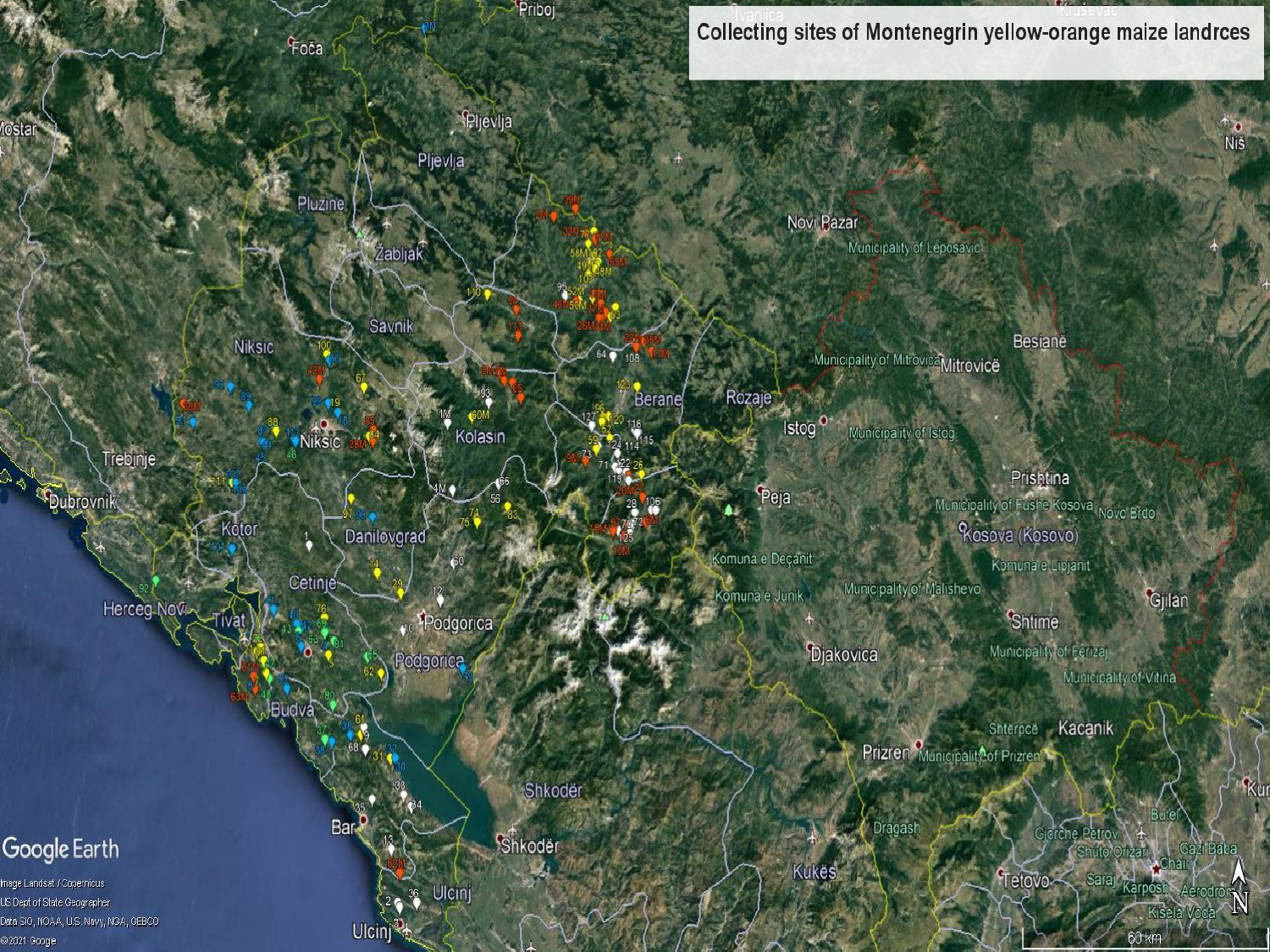

Supplement: Supplementary file 1 [file plants-10-01503-s001.zip › File S1.pptx]
